# Redetermination of bis­(2-formyl­phenolato-κ^2^ *O*,*O*′)nickel(II) as bis­[2-(imino­meth­yl)phenolato-κ^2^ *N*,*O*′]nickel(II)

**DOI:** 10.1107/S160053680905483X

**Published:** 2009-12-24

**Authors:** Sylvain Bernès

**Affiliations:** a DEP Facultad de Ciencias Químicas, UANL, Guerrero y Progreso S/N, Col. Treviño, 64570 Monterrey, NL, Mexico

## Abstract

The crystal structure of bis­(2-formyl­phenolato-κ^2^ *O*,*O*′)nickel(II), [Ni(C_7_H_5_O_2_)_2_], a square-planar centrosymmetric complex, has been reported previously [Li & Chen (2006). *Acta Cryst*. E**62**, m1038–m1039]. However, a number of warning signs allows the assumption that the carbonyl group in the salicylaldehydate ligand of the claimed complex is incorrect. The crystal structure was therefore redetermined on basis of the originally deposited structure factors. After substituting the carbonyl O atom by an N atom, the model can be completed with an imine H atom, which was clearly discernible in a difference map. The resulting model, corresponding to bis­[2-(imino­meth­yl)phenolato-κ^2^ *N*,*O*′]nickel(II), [Ni(C_7_H_6_NO)_2_], converges well and none of the previous structural alerts remains. This reinter­pretation is also consistent with the published synthesis, which was carried out using salicylaldehyde in the presence of aqueous NH_3_. The reinter­preted structure is virtually identical to earlier reports dealing with this bis-iminato Ni^II^ complex.

## Related literature

For the original structure, see: Li & Chen (2006 ▶). For the tools used for reinter­pretation, see: Bruno *et al.* (2004 ▶); Spek (2009 ▶); Hirshfeld (1976 ▶). For earlier reports on the synthesis and crystal structure of bis­(2-salicylideneiminato-*κ* ^2^ *N*,*O*′)nickel(II), see: Mustafa *et al.* (2001 ▶); Simonsen & Pfluger (1957 ▶); Stewart & Lingafelter (1959 ▶); Kamenar *et al.* (1990 ▶); De *et al.* (1999 ▶).
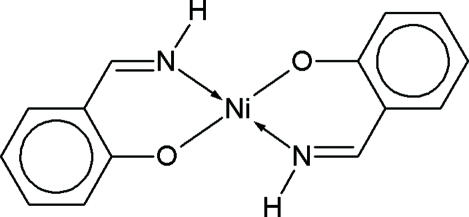


## Experimental

### 

#### Crystal data

[Ni(C_7_H_6_NO)_2_]
*M* *_r_* = 298.97
Monoclinic, 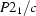

*a* = 12.934 (3) Å
*b* = 5.827 (1) Å
*c* = 8.108 (2) Å
β = 95.67 (3)°
*V* = 608.1 (2) Å^3^
*Z* = 2
Mo *K*α radiation
μ = 1.59 mm^−1^
*T* = 293 K
0.24 × 0.21 × 0.16 mm

#### Data collection

Siemens R3m diffractometer
Absorption correction: ψ scan (Kopfmann & Huber, 1968 ▶) *T*_min_ = 0.688, *T*_max_ = 0.774
1224 measured reflections
1224 independent reflections
856 reflections with *I* > 2σ(*I*)
2 standard reflections every 200 reflections  intensity decay: none

#### Refinement

*R*[*F* ^2^ > 2σ(*F* ^2^)] = 0.041
*wR*(*F* ^2^) = 0.091
*S* = 0.95
1224 reflections
91 parameters
H atoms treated by a mixture of independent and constrained refinement
Δρ_max_ = 0.54 e Å^−3^
Δρ_min_ = −0.29 e Å^−3^

### 

Data collection: *XSCANS* (Siemens, 1990 ▶); cell refinement: *XSCANS*; data reduction: *SHELXTL-Plus* (Sheldrick, 2008 ▶); program(s) used to solve structure: *WinGX* (Farrugia, 1999 ▶); program(s) used to refine structure: *SHELXTL-Plus*; molecular graphics: *SHELXTL-Plus*; software used to prepare material for publication: *SHELXTL-Plus*.

## Supplementary Material

Crystal structure: contains datablocks I, global. DOI: 10.1107/S160053680905483X/wm2259sup1.cif

Structure factors: contains datablocks I. DOI: 10.1107/S160053680905483X/wm2259Isup2.hkl

Additional supplementary materials:  crystallographic information; 3D view; checkCIF report

## Figures and Tables

**Fig. 1.**
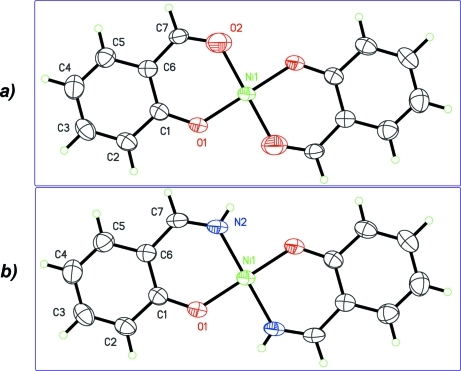
Two ORTEP-style views of the original (a) and reinterpreted (b) complexes. Displacement ellipsoids for non-H atoms are shown at the 60% probability level. Symmetry code for non-labeled atoms: 1 - x, 1 - y, 1 - z.

## supplementary crystallographic information

### Comment

The increasingly fast routine structure determination, associated with the growing availability of CCD-based diffractometers, produced a high number of deposited structures in the last decade. Although the peer-review process is now, at least in part, automated through powerful checking programs, the possibility to see the rate of deposition going out of control is real. A growing concomitant concern is related to the fact that the number of structures of questionable quality will necessarily be increased in a near future. Strategies avoiding the deposition of wrong structures are definitively the best approach, compared to those based on post-deposition data mining, which are time consuming, and depend strongly on how databases are formatted.

The following example shows how a couple of freely available checking tools can detect the wrong assignment of a functional group with a different, isoelectronic group, for instance SH *vs*. Cl, CH_3_*vs*. F, *etc*, with a high degree of confidence.

The crystal structure of the centrosymmetric complex bis(2-formylphenolato-*κ*^2^*O*,*O*')nickel(II) was originally reported by Li & Chen (2006) in space group *P*2_1_/*c*, with sensible key indicators. The *ORTEP* plot of the complex (Fig. 1) shows however a large displacement parameter for the carbonyl O atom (O2) in the salicylaldehydate ligand, compared to those of other atoms. On the other hand, *PLATON* (Spek, 2009) detects a significant Hirshfeld rigid bond test violation (9.5 s.u.) for this CO bond (Hirshfeld, 1976). Finally, a check for the geometry using *Mogul* (version 1.1.3; Bruno *et al.*, 2004) alerts on an unusually small C—CO angle, 124.5 (4)°, while the expected value from 34 hit fragments retrieved from the database is 128.2 (18)°. The resulting *z*-score, 2.056, may be related to an actual problem with the assignment of this functional group.

It is worth noting that none of the above described alerts is a clear indication of a wrongly assigned scattering factor. However, the *combined* Hirshfeld and *Mogul* alerts for a single CO functional group should be regarded as a worrying signal about the claimed structure, and thus should be carefully checked. In the present case, all becomes clear from the synthetic route used for the Ni^II^ complex preparation: since salicylaldehyde is used as starting material in hot ethanol and aqueous ammonia (0.5 *M*) that was added to adjust the pH to 7, the imine should be formed readily, which then reacts with Ni^II^ (Mustafa *et al.*, 2001). The crystal collected in the original study was thus more likely to be bis(2-salicylideneiminato-*κ*^2^*O*,*O*')nickel(II) rather than the claimed salicylaldehydate complex.

Using the deposited structure factors of the original publication by Li & Chen (2006), this hypothesis has been corroborated. Starting from the original set of coordinates, the model was modified substituting atom O2 by an N atom, and completed by interpreting the highest peak found in a difference map as an imine H atom (H2), which was refined freely (Fig. 1). The refinement converges well, and the residual is reduced to *R*_1_ = 0.041, while the original model converged to *R*_1_ = 0.046. In addition, all former structural alerts are no longer present in the reinterpreted model: for instance, *Mogul* affords a *z*-score of 0.42 for the C—CN angle, based on 16 fragment hits. All bond lengths and angles are in the expected ranges in the final model. Finally, the structure obtained after reinterpretation of the model is virtually identical to that documented in earlier publications (Simonsen & Pfluger, 1957; Stewart & Lingafelter, 1959; Kamenar *et al.*, 1990; De *et al.*, 1999).

### Experimental

For the originally reported synthesis, see: Li & Chen (2006)

### Refinement

Deposited CIF and structure factors files were downloaded from the web and exported to *SHELX* compatible files using the *WinGX* facilities (Version 1.80.05, Farrugia, 1999). After substituting O2 by N2, the model was refined. The highest peak in a difference map, found at *ca*. 1 Å from N2, was interpreted as an H atom, and refined freely, with *U*_iso_ = 0.08 Å^2^. Other parameters were kept as in the original publication, except for extinction correction, which was not applied.

### Crystal data

**Table:**

[Ni(C_7_H_6_NO)_2_]	*F*(000) = 308
*M**_r_* = 298.97	*D*_x_ = 1.633 Mg m^−^^3^
Monoclinic, *P*2_1_/*c*	Mo *K*α radiation, λ = 0.71073 Å
Hall symbol: -P 2ybc	Cell parameters from 25 reflections
*a* = 12.934 (3) Å	θ = 6.5–15°
*b* = 5.827 (1) Å	µ = 1.59 mm^−^^1^
*c* = 8.108 (2) Å	*T* = 293 K
β = 95.67 (3)°	Block, red
*V* = 608.1 (2) Å^3^	0.24 × 0.21 × 0.16 mm
*Z* = 2	

### Data collection

**Table:**

Siemens R3m diffractometer	856 reflections with *I* > 2σ(*I*)
Radiation source: fine-focus sealed tube	*R*_int_ = 0.0000
graphite	θ_max_ = 27.0°, θ_min_ = 3.2°
ω scans	*h* = −16→16
Absorption correction: ψ scan (Kopfmann & Huber, 1968)	*k* = 0→7
*T*_min_ = 0.688, *T*_max_ = 0.774	*l* = 0→10
1224 measured reflections	2 standard reflections every 200 reflections
1224 independent reflections	intensity decay: none

### Refinement

**Table:**

Refinement on *F*^2^	Primary atom site location: See text
Least-squares matrix: full	Secondary atom site location: difference Fourier map
*R*[*F*^2^ > 2σ(*F*^2^)] = 0.041	Hydrogen site location: inferred from neighbouring sites
*wR*(*F*^2^) = 0.091	H atoms treated by a mixture of independent and constrained refinement
*S* = 0.95	*w* = 1/[σ^2^(*F*_o_^2^) + (0.0158*P*)^2^ + 1.3629*P*] where *P* = (*F*_o_^2^ + 2*F*_c_^2^)/3
1224 reflections	(Δ/σ)_max_ < 0.001
91 parameters	Δρ_max_ = 0.54 e Å^−^^3^
0 restraints	Δρ_min_ = −0.29 e Å^−^^3^
0 constraints	

### Fractional atomic coordinates and isotropic or equivalent isotropic displacement parameters (Å^2^)

**Table:**

	*x*	*y*	*z*	*U*_iso_*/*U*_eq_	
Ni1	0.5000	0.5000	0.5000	0.0286 (2)	
O1	0.59659 (18)	0.6557 (4)	0.6384 (3)	0.0365 (6)	
N2	0.5867 (2)	0.2527 (5)	0.4760 (4)	0.0327 (7)	
H2	0.566 (4)	0.155 (10)	0.426 (6)	0.080*	
C1	0.6925 (3)	0.5968 (6)	0.6852 (4)	0.0273 (7)	
C2	0.7541 (3)	0.7483 (6)	0.7889 (4)	0.0322 (8)	
H2A	0.7255	0.8854	0.8217	0.080*	
C3	0.8555 (3)	0.6969 (7)	0.8424 (4)	0.0380 (9)	
H3A	0.8951	0.8018	0.9080	0.080*	
C4	0.9001 (3)	0.4889 (8)	0.7996 (4)	0.0425 (9)	
H4A	0.9681	0.4524	0.8392	0.080*	
C5	0.8416 (3)	0.3408 (7)	0.6985 (4)	0.0367 (9)	
H5A	0.8714	0.2044	0.6669	0.080*	
C6	0.7383 (3)	0.3887 (6)	0.6412 (4)	0.0281 (7)	
C7	0.6807 (3)	0.2243 (6)	0.5385 (4)	0.0302 (8)	
H7A	0.7136	0.0877	0.5155	0.080*	

### Atomic displacement parameters (Å^2^)

**Table:**

	*U*^11^	*U*^22^	*U*^33^	*U*^12^	*U*^13^	*U*^23^
Ni1	0.0320 (3)	0.0210 (3)	0.0320 (3)	0.0036 (3)	−0.0010 (2)	−0.0070 (3)
O1	0.0379 (14)	0.0282 (14)	0.0416 (14)	0.0053 (11)	−0.0051 (11)	−0.0141 (11)
N2	0.0400 (17)	0.0209 (16)	0.0368 (17)	0.0032 (14)	0.0012 (13)	−0.0101 (13)
C1	0.0332 (18)	0.0237 (16)	0.0248 (16)	−0.0026 (15)	0.0022 (14)	0.0017 (14)
C2	0.045 (2)	0.0231 (18)	0.0279 (17)	−0.0029 (16)	0.0023 (15)	0.0016 (14)
C3	0.044 (2)	0.035 (2)	0.035 (2)	−0.0122 (18)	−0.0021 (16)	0.0012 (16)
C4	0.0359 (18)	0.045 (2)	0.0454 (19)	0.000 (2)	−0.0030 (15)	0.005 (2)
C5	0.041 (2)	0.034 (2)	0.0349 (19)	0.0044 (17)	0.0024 (15)	0.0006 (16)
C6	0.0351 (18)	0.0251 (18)	0.0243 (16)	0.0006 (15)	0.0041 (14)	0.0015 (14)
C7	0.0413 (19)	0.0195 (17)	0.0304 (17)	0.0045 (15)	0.0054 (15)	−0.0028 (14)

### Geometric parameters (Å, °)

**Table:**

Ni1—O1^i^	1.835 (2)	C2—H2A	0.9300
Ni1—O1	1.835 (2)	C3—C4	1.401 (6)
Ni1—N2	1.848 (3)	C3—H3A	0.9300
Ni1—N2^i^	1.848 (3)	C4—C5	1.366 (5)
O1—C1	1.307 (4)	C4—H4A	0.9300
N2—C7	1.280 (5)	C5—C6	1.399 (5)
N2—H2	0.73 (5)	C5—H5A	0.9300
C1—C6	1.410 (5)	C6—C7	1.430 (5)
C1—C2	1.410 (5)	C7—H7A	0.9300
C2—C3	1.373 (5)		
			
O1^i^—Ni1—O1	180.000 (1)	C2—C3—C4	121.0 (3)
O1^i^—Ni1—N2	86.19 (11)	C2—C3—H3A	119.5
O1—Ni1—N2	93.81 (11)	C4—C3—H3A	119.5
O1^i^—Ni1—N2^i^	93.81 (11)	C5—C4—C3	118.6 (3)
O1—Ni1—N2^i^	86.19 (11)	C5—C4—H4A	120.7
N2—Ni1—N2^i^	180.00 (18)	C3—C4—H4A	120.7
C1—O1—Ni1	128.1 (2)	C4—C5—C6	121.8 (4)
C7—N2—Ni1	128.4 (2)	C4—C5—H5A	119.1
C7—N2—H2	114 (4)	C6—C5—H5A	119.1
Ni1—N2—H2	118 (4)	C5—C6—C1	119.9 (3)
O1—C1—C6	124.2 (3)	C5—C6—C7	119.0 (3)
O1—C1—C2	118.2 (3)	C1—C6—C7	121.1 (3)
C6—C1—C2	117.6 (3)	N2—C7—C6	124.3 (3)
C3—C2—C1	121.1 (3)	N2—C7—H7A	117.9
C3—C2—H2A	119.4	C6—C7—H7A	117.9
C1—C2—H2A	119.4		
			
N2—Ni1—O1—C1	3.4 (3)	C3—C4—C5—C6	−1.9 (6)
N2^i^—Ni1—O1—C1	−176.6 (3)	C4—C5—C6—C1	1.3 (5)
O1^i^—Ni1—N2—C7	178.7 (3)	C4—C5—C6—C7	−178.7 (3)
O1—Ni1—N2—C7	−1.3 (3)	O1—C1—C6—C5	−179.6 (3)
Ni1—O1—C1—C6	−3.3 (5)	C2—C1—C6—C5	−0.9 (5)
Ni1—O1—C1—C2	178.0 (2)	O1—C1—C6—C7	0.4 (5)
O1—C1—C2—C3	−179.9 (3)	C2—C1—C6—C7	179.1 (3)
C6—C1—C2—C3	1.3 (5)	Ni1—N2—C7—C6	−0.9 (5)
C1—C2—C3—C4	−1.9 (5)	C5—C6—C7—N2	−178.2 (3)
C2—C3—C4—C5	2.2 (6)	C1—C6—C7—N2	1.8 (5)

Symmetry codes: (i) −*x*+1, −*y*+1, −*z*+1.

## References

[bb1] Bruno, I. J., Cole, J. C., Kessler, M., Luo, J., Motherwell, W. D. S., Purkis, L. H., Smith, B. R., Taylor, R., Cooper, R. I., Harris, S. E. & Orpen, A. G. (2004). *J. Chem. Inf. Comput. Sci* **44**, 2133–2144. 10.1021/ci049780b 15554684

[bb2] De, R. L., Banerjee, I., Samanta, C. & Mukherjee, A. K. (1999). *Indian J. Chem. Sect. A*, **38**, 373–376.

[bb3] Farrugia, L. J. (1999). *J. Appl. Cryst.* **32**, 837–838.

[bb4] Hirshfeld, F. L. (1976). *Acta Cryst.* A**32**, 239–244.

[bb5] Kamenar, B., Kaitner, B., Ferguson, G. & Waters, T. N. (1990). *Acta Cryst.* C**46**, 1920–1923.

[bb6] Kopfmann, G. & Huber, R. (1968). *Acta Cryst.* A**24**, 348–351.

[bb7] Li, Y.-G. & Chen, H.-J. (2006). *Acta Cryst.* E**62**, m1038–m1039.

[bb8] Mustafa, I. A., Taki, M. H. & Al-Allaf, T. A. K. (2001). *Asian J. Chem* **13**, 1039–1047.

[bb9] Sheldrick, G. M. (2008). *Acta Cryst.* A**64**, 112–122. 10.1107/S0108767307043930 18156677

[bb10] Siemens (1990). *XSCANS User’s Manual* Siemens Analytical X-ray Instruments Inc., Madison, Wisconsin, USA.

[bb11] Simonsen, S. H. & Pfluger, C. E. (1957). *Acta Cryst.* **10**, 471.

[bb12] Spek, A. L. (2009). *Acta Cryst.* D**65**, 148–155. 10.1107/S090744490804362X PMC2631630 19171970

[bb13] Stewart, J. M. & Lingafelter, E. C. (1959). *Acta Cryst* **12**, 842–845.

